# Inhibition of Potassium Channels Affects the Ability of Pig Spermatozoa to Elicit Capacitation and Trigger the Acrosome Exocytosis Induced by Progesterone

**DOI:** 10.3390/ijms22041992

**Published:** 2021-02-17

**Authors:** Federico Noto, Sandra Recuero, Julián Valencia, Beatrice Saporito, Domenico Robbe, Sergi Bonet, Augusto Carluccio, Marc Yeste

**Affiliations:** 1 Biotechnology of Animal and Human Reproduction (TechnoSperm), Institute of Food and Agricultural Technology, University of Girona, ES-17003 Girona, Spain; fnoto83@gmail.com (F.N.); sandra.recuero@udg.edu (S.R.); sergi.bonet@udg.edu (S.B.); 2 Unit of Cell Biology, Department of Biology, Faculty of Sciences, University of Girona, ES-17003 Girona, Spain; jvalencia21@uan.edu.co; 3 Faculty of Veterinary Medicine, University of Teramo, Località Piano D’Accio, IT-64100 Teramo, Italy; beatrice.saporito.93@gmail.com (B.S.); drobbe@unite.it (D.R.); acarluccio@unite.it (A.C.); 4 University Antonio Nariño, Calle 53 #9-35, Popayán CO-190002, Colombia

**Keywords:** pigs, spermatozoa, capacitation, potassium channels, quinine, paxilline

## Abstract

During capacitation, sperm undergo a myriad of changes, including remodeling of plasma membrane, modification of sperm motility and kinematic parameters, membrane hyperpolarization, increase in intracellular calcium levels, and tyrosine phosphorylation of certain sperm proteins. While potassium channels have been reported to be crucial for capacitation of mouse and human sperm, their role in pigs has not been investigated. With this purpose, sperm samples from 15 boars were incubated in capacitation medium for 300 min with quinine, a general blocker of potassium channels (including voltage-gated potassium channels, calcium-activated potassium channels, and tandem pore domain potassium channels), and paxilline (PAX), a specific inhibitor of calcium-activated potassium channels. In all samples, acrosome exocytosis was induced after 240 min of incubation with progesterone. Plasma membrane and acrosome integrity, membrane lipid disorder, intracellular calcium levels, mitochondrial membrane potential, and total and progressive sperm motility were evaluated after 0, 120, and 240 min of incubation, and after 5, 30, and 60 min of progesterone addition. Although blocking potassium channels with quinine and PAX prevented sperm to elicit in vitro capacitation by impairing motility and mitochondrial function, as well as reducing intracellular calcium levels, the extent of that inhibition was larger with quinine than with PAX. Therefore, while our data support that calcium-activated potassium channels are essential for sperm capacitation in pigs, they also suggest that other potassium channels, such as the voltage-gated, tandem pore domain, and mitochondrial ATP-regulated ones, are involved in that process. Thus, further research is needed to elucidate the specific functions of these channels and the mechanisms underlying its regulation during sperm capacitation.

## 1. Introduction

Ion channels are crucial for sperm physiology and are involved in the sperm transport throughout the male and female reproductive tracts, epididymal maturation, motility activation, chemotaxis and thermotaxis, capacitation, and acrosome reaction [1,2,3,4,5]. These ion channels allow the rapid movement of millions of ions flowing per second across the lipid bilayer and, together with ion pumps, they allow sperm cells to regulate concentration gradients, which affects intracellular calcium levels, cell volume, and pH [6,7,8,9]. Chloride, Cystic fibrosis transmembrane conductance regulator (CFTR), potassium, and calcium channels are the main ion channels in mammalian spermatozoa; specifically, potassium channels are involved in the regulation of sperm volume and underlie the hyperpolarization of sperm plasma membrane, which is involved in the regulation of sperm motility and acrosome reaction [2,10,11].

Potassium channels have been detected in spermatogenic cells and mature spermatozoa [12] and, based on its functionality and structure [13], they are classified into four classes [13]: (a) Voltage-gated potassium channels (Kv); (b) calcium-activated potassium channels (KCa); (c) inwardly rectifying potassium channels (Kir); and (d) tandem pore domain potassium channels (K_2P_). The first two types can have overlapping properties as happens with SLO3, a voltage-gated potassium channel modulated by intracellular pH [14,15], and with SLO1, a calcium voltage-gated potassium channel [4]. While the role of ligand-activated potassium channels during pig sperm capacitation has not been studied much, these ion channels have been found to play an instrumental role for sperm membrane hyperpolarization in humans and mice [16,17,18,19,20].

Upon ejaculation, mammalian spermatozoa are not yet ready to fertilize the oocyte, but rather need to undergo a series of changes that occur within the female reproductive tract and are known as sperm capacitation [21,22,23]. Elucidating the role of potassium channels during capacitation of pig spermatozoa may be conducted through the use of blocking agents, in a similar fashion to previous studies [4,24,25]. Quinine is an alkaloid extracted from cinchona bark, which was the first effective antimalarial drug [26]. Quinine inhibits many K^+^ channels including KCa, Kv (e.g., Kv2.2, which are encoded by KCNB2) and K_2P_ channels (e.g., Kv18.1, encoded by KCNK18), which have been reported to be involved in the regulation of cell volume [27,28], including mammalian sperm [29]. While incubation of human sperm with quinine has been found to increase their volume and induce kinematic alterations, which are crucial for sperm transport, including penetration and migration throughout the cervical mucus [11,30], there is no information about their implication in pig sperm capacitation. On the other hand, another inhibitor is paxilline, which specifically blocks KCa channels (e.g., KCa1.1, encoded by KCNMA1) [31,32]. Neither quinine nor paxilline have any effect on Na^+^ and Ca^2+^ channels.

Against this background, this study sought to investigate the role of calcium-activated potassium channels during in vitro capacitation and acrosome reaction of pig spermatozoa. With this purpose, we tested the effects of general (quinine) and specific (paxilline) inhibitors of calcium-activated potassium channels at two concentrations (0.1 mM and 1 mM) on the integrity and lipid disorder of plasma membrane, acrosome exocytosis, intracellular calcium levels, mitochondrial membrane potential, and sperm motility. Our hypothesis is that because calcium-activated potassium channels have been demonstrated to be crucial for mammalian sperm physiology, their inhibition should modify the sperm ability to elicit in vitro capacitation and trigger the acrosome reaction induced by progesterone.

## 2. Results

### 2.1. Effects of Quinine and Paxilline on Plasma Membrane Integrity

**Figure 1:**
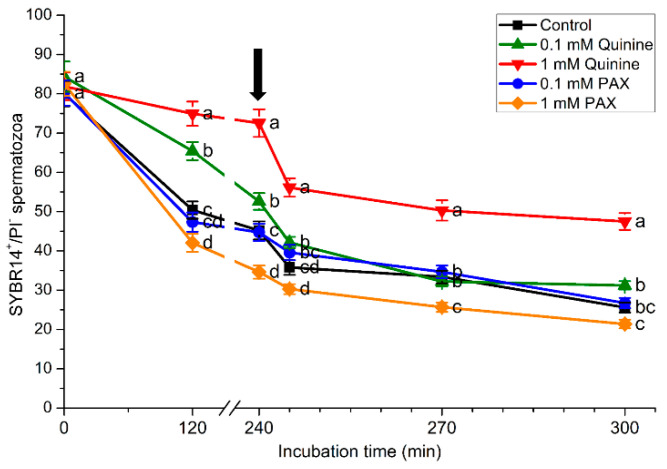
Percentages of spermatozoa with an intact plasma membrane (SYBR14^+^/PI^−^) during in vitro capacitation and progesterone-induced acrosomal exocytosis (300 min) in the control and treatments containing 0.1 mM quinine, 1 mM quinine, 0.1 mM paxilline (PAX), or 1 mM PAX. Black arrow indicates the time at which 10 μg/mL progesterone was added to induce acrosomal exocytosis (i.e., 240 min). Different letters (a–d) indicate significant (*p* < 0.05) differences between treatments at a given time point. Data are shown as mean ± SEM for 15 independent experiments.

Figure 1 shows the effects of quinine and PAX on plasma membrane integrity during sperm capacitation. Incubation of pig sperm with capacitation medium led, as expected, to a reduction of sperm membrane integrity (SYBR14^+^/PI^−^) in all treatments (*p* < 0.05). However, when quinine was present, the extent of that reduction was lower in a dose-dependent manner. Therefore, after 120 min and 240 min of incubation and after progesterone addition, percentages of sperm with an intact plasma membrane in the treatment containing 1 mM quinine were significantly higher (*p* < 0.05) than in the control. Furthermore, after 120 and 240 min of incubation and after 5 min of progesterone addition (245 min), percentages of sperm with an intact plasma membrane in the treatment containing 0.1 mM quinine were significantly higher (*p* < 0.05) than in the control. Conversely, the presence of PAX either had no effect on plasma membrane integrity (0.1 mM) or significantly (*p* < 0.05) reduced the percentages of spermatozoa with an intact plasma membrane (1 mM) after 240 min of incubation and after 30 min of progesterone addition (270 min), when compared to the control.

### 2.2. Effects of Quinine and Paxilline on Acrosome Integrity

**Figure 2:**
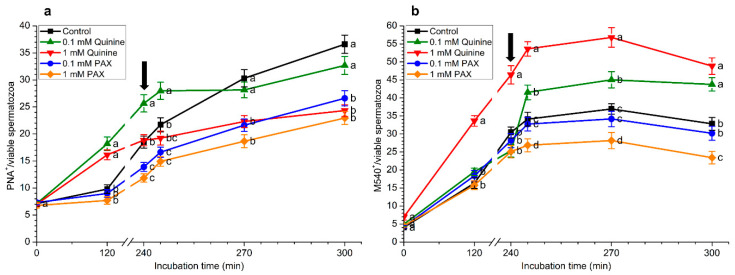
(**a**) Percentages of spermatozoa with an exocytosed acrosome (PNA^−^) within the viable sperm population and (**b**) percentages of spermatozoa with high membrane lipid disorder (M540^−^) within the viable sperm population during in vitro capacitation and progesterone-induced acrosomal exocytosis (300 min) in the presence of 0.1 mM quinine, 1 mM quinine, 0.1 mM paxilline, (PAX), and 1 mM PAX. Black arrow indicates the time at which 10 μg/mL progesterone was added to induce acrosomal exocytosis (i.e., 240 min). Different letters (a–c) mean significant (*p* < 0.05) differences between treatments at a given time point. Data are shown as mean ± SEM for 15 independent experiments.

Percentages of viable spermatozoa with an intact acrosome membrane (PNA^+^/EthD-1^−^) significantly (*p* < 0.05) decreased throughout incubation in capacitation medium (Figure S1a). This decrease was more apparent in the control and samples incubated with 0.1 mM quinine, 0.1 mM PAX, and 1 mM PAX than in those containing 1 mM quinine. After 30 min and 60 min of the addition of progesterone (i.e., 270 min and 300 min), percentages of viable spermatozoa with an intact acrosome were significantly (*p* < 0.05) higher in samples incubated with 1 mM quinine than in the control and in samples containing 0.1 mM quinine, 0.1 mM PAX, or 1 mM PAX. After 60 min of progesterone addition (300 min), all samples containing quinine or PAX showed significantly (*p* < 0.05) higher percentages of viable spermatozoa with an intact acrosome membrane than the control. Furthermore, spermatozoa showing an exocytosed acrosome within the viable sperm population (PNA^−^/viable spermatozoa) were significantly (*p* < 0.05) lower in the treatment containing 1 mM quinine than in the control and samples with 0.1 mM quinine, 0.1 mM PAX, or 1 mM PAX after 60 min of progesterone addition (300 min; Figure 2a).

### 2.3. Effects of Quinine and Paxilline on Membrane Lipid Disorder

Figure 2b, Figures S1b and S2 show the effects of blocking potassium channels with quinine or PAX on the percentages of spermatozoa with low membrane lipid disorder. Although there was a significant (*p* < 0.05) decrease in the percentages of viable spermatozoa with low membrane lipid disorder (M540^−^/YO-PRO-1^−^) throughout in vitro capacitation and after progesterone addition, no significant (*p* > 0.05) differences between treatments and the control were observed (Figure S1b).

In contrast, and as depicted in Figure 2b, incubation with capacitation medium significantly (*p* < 0.05) increased the percentages of spermatozoa with high membrane lipid disorder (M540^+^) within the viable sperm population. However, these percentages were significantly (*p* < 0.05) higher in the presence of 1 mM quinine than in the control after 120 min of incubation and until the end of the incubation period. Conversely, the percentages of spermatozoa with high membrane lipid disorder (M540^+^) within the viable sperm population in the treatment containing 1 mM PAX were significantly (*p* < 0.05) lower than in the control and the other treatments after 5 min, 30 min, and 60 min of progesterone addition (i.e., 245 min, 270 min, and 300 min).

### 2.4. Effects of Quinine and Paxilline on Intracellular Calcium Levels

**Figure 3:**
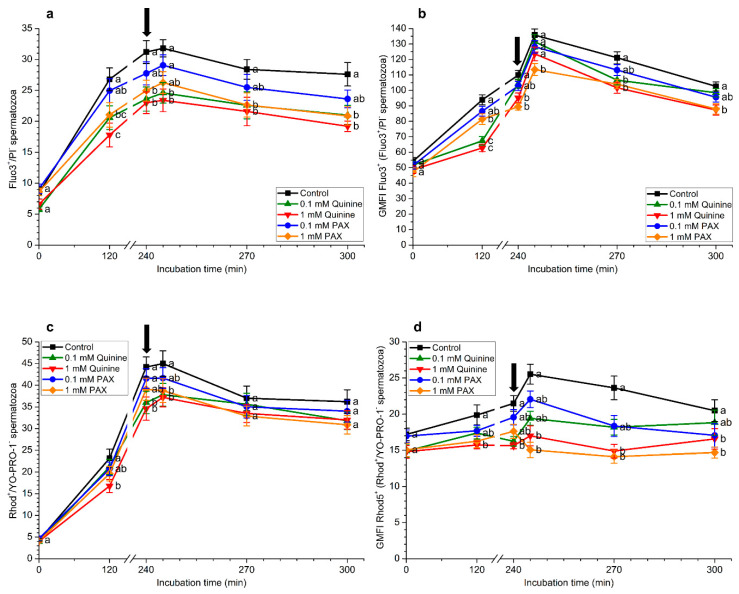
(**a**) Percentages of viable spermatozoa with high intracellular calcium levels evaluated with Fluo3 (Fluo3^+^/PI^−^), (**b**) geometric mean of fluorescence intensity (GMFI) of Fluo3 in Fluo3^+^/PI^−^ sperm population, (**c**) percentages of viable spermatozoa with high intracellular calcium levels evaluated with Rhod5 (Rhod5^+^/YO-PRO-1^−^) and (**d**) geometric mean of fluorescence intensity (GMFI) of Rhod5 in Rhod5^+^/YO-PRO-1^−^ sperm population during in vitro capacitation and progesterone-induced acrosomal exocytosis (300 min) in the presence of 0.1 mM quinine, 1 mM quinine, 0.1 mM paxilline (PAX), and 1 mM PAX. Black arrow indicates the time at which 10 μg/mL progesterone was added to induce acrosomal exocytosis (i.e., 240 min). Different letters (a,b) mean significant (*p* < 0.05) differences between treatments at a given time point. Data are shown as mean ± SEM for 15 independent experiments.

Figure 3a shows the effects of quinine and PAX on the percentages of viable spermatozoa with high intracellular calcium levels stained by Fluo3 (Fluo3^+^/PI^−^). In all treatments, incubation in capacitation medium significantly (*p* < 0.05) increased the percentages of Fluo3^+^/PI^−^ spermatozoa. The extent of that increase was, however, significantly (*p* < 0.05) higher in the control than in the treatments containing 0.1 mM quinine, 1 mM quinine, or 1 mM PAX after 120 min and 240 min incubation and after the addition of progesterone. No significant differences (*p* > 0.05) between treatments containing 0.1 quinine, 1 mM quinine, or 1 mM PAX were observed, except after 5 min of progesterone addition (245 min).

As Figure 3b depicts, geometric mean of Fluo3^+^-intensity in the Fluo3^+^/PI^−^ sperm population was significantly (*p* < 0.05) higher in the control than in treatments containing 1 mM quinine and 1 mM PAX after 120 min and 240 min of incubation and after progesterone addition. Furthermore, geometric mean of Fluo3^+^-intensity in the Fluo3^+^/PI^−^ sperm population was also significantly (*p* < 0.05) higher in the control than in the treatment containing 0.1 mM quinine at 120 min and after 30 min of the addition of progesterone (270 min).

In a similar fashion to that described for Fluo3-staining and as Figure 3c shows, percentages of viable spermatozoa with high intracellular calcium levels stained by Rhod5 (Rhod5^+^/YO-PRO-1^−^) significantly (*p* < 0.05) increased following incubation with capacitation medium. Again, the control presented significantly (*p* < 0.05) higher percentages of Rhod5^+^/YO-PRO-1^−^ spermatozoa than the treatment containing 1 mM quinine after 120 min and 240 min of incubation and 5 min after progesterone addition (245 min), and higher than the treatment with 1 mM PAX after 5 min of progesterone addition (245 min).

In the case of geometric mean of Rhod5^+^-intensity in the Rhod5^+^/YO-PRO-1^−^ sperm population (Figure 3d), spermatozoa incubated in the control medium showed significantly (*p* < 0.05) higher values of this parameter than those containing 1 mM quinine or 1 mM PAX after 240 min of incubation and following progesterone addition (i.e., 245, 270, and 300 min).

### 2.5. Effects of Quinine and Paxilline on Mitochondrial Membrane Potential

**Figure 4:**
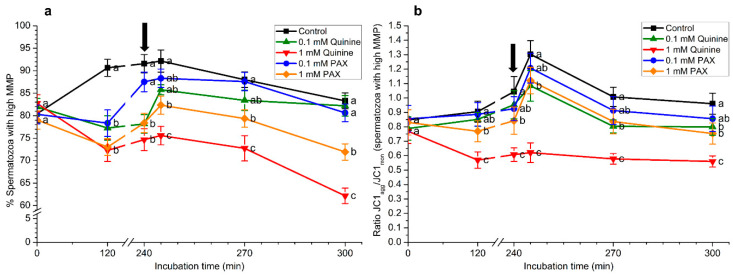
(**a**) Percentages of spermatozoa with high mitochondrial membrane potential (MMP) and (**b**) their JC1_agg_/JC1_mon_ ratios during in vitro capacitation and progesterone-induced acrosomal exocytosis (300 min) in the presence of 0.1 mM quinine, 1 mM quinine, 0.1 mM paxilline (PAX), and 1 mM paxilline. Black arrow indicates the time at which 10 μg/mL progesterone was added to induce acrosomal exocytosis (i.e., 240 min). Different letters (a–c) mean significant (*p* < 0.05) differences between treatments at a given time point. Data are shown as mean ± SEM for 15 independent experiments.

As shown in Figure 4a and Figure S3, after 120 min of incubation and until the end of the experiment, percentages of spermatozoa with high mitochondrial membrane potential (MMP) were significantly (*p* < 0.05) higher in the control than in the treatments containing 1 mM quinine and 1 mM PAX. In addition, percentages of spermatozoa with high MMP were significantly (*p* < 0.05) higher in the treatment containing 0.1 mM quinine than in that with 1 mM quinine after progesterone addition (i.e., 245, 270 min, and 300 min). Moreover, the percentages of spermatozoa with high MMP were significantly (*p* < 0.05) higher in the treatment containing 0.1 mM PAX than in that with 1 mM PAX at 120 min, and after 30 min and 60 min of progesterone addition (270 min and 300 min).

The results observed for the percentages of spermatozoa with high MMP were similar to those found in their JC1_agg_/JC1_mon_ ratios (Figure 4b). In effect, these ratios were significantly (*p* < 0.05) lower in the control than in the treatments containing 1 mM quinine or 1 mM PAX at 120 min and 240 min, and after the addition of progesterone. Furthermore, JC1_agg_/JC1_mon_ ratios of the sperm population with high MMP were significantly (*p* < 0.05) lower in the treatment containing 0.1 mM quinine than in the control at 240 min and after 30 min of progesterone addition (270 min). In contrast, no significant differences between the control and the treatment containing 0.1 mM PAX were observed.

### 2.6. Effects of Quinine and Paxilline on Sperm Motility

**Figure 5:**
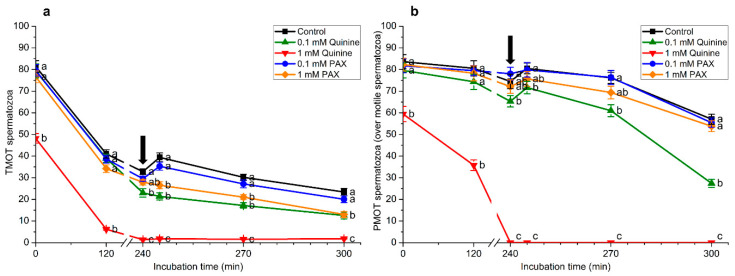
(**a**) Percentages of total motile (TMOT) and (**b**) of progressively motile spermatozoa (calculated over motile cells; PMOT) during in vitro capacitation and progesterone-induced acrosomal exocytosis (300 min) in the presence of 0.1 mM quinine, 1 mM quinine, 0.1 mM paxilline (PAX), and 1 mM paxilline. Black arrow indicates the time at which 10 μg/mL progesterone was added to induce acrosomal exocytosis (i.e., 240 min). Different letters (a–c) mean significant (*p* < 0.05) differences between treatments at a given time point. Data are shown as mean ± SEM for 15 independent experiments.

Inhibition of potassium channels with 1 mM quinine led to a dramatic drop in the percentages of total (Figure 5a) and progressively motile spermatozoa (calculated over motile cells) at the beginning of the experiment (Figure 5b). Thus, percentages of total and progressively motile spermatozoa were significantly (*p* < 0.05) lower in the treatment containing 1 mM quinine than in the control and the other treatments (0.1 mM quinine, 0.1 mM PAX, and 1 mM PAX) throughout the entire incubation period and following the addition of progesterone. Moreover, percentages of total and progressively motile spermatozoa were significantly (*p* < 0.05) higher in the control than in the treatment containing 0.1 mM quinine after progesterone addition (i.e., 245, 270, and 300 min). On the other hand, no significant differences between the control and treatments containing 0.1 mM and 1 mM PAX were observed in the percentages of progressively motile spermatozoa throughout the entire incubation period, which contrasted with that observed for the percentages of total motile spermatozoa in the treatment with 1 mM PAX after the addition of progesterone (245, 270, and 300 min).

## 3. Discussion

While calcium-activated potassium channels are known to have a crucial role for mouse [17,31] and human sperm physiology [19,20], their role during pig sperm capacitation has not yet been studied. Taking this into account, the present study aimed at determining the contribution of potassium channels during in vitro capacitation and progesterone-induced acrosome exocytosis of pig spermatozoa through their inhibition with quinine, an inhibitor of a wide range of potassium channels (including voltage-gated potassium channels, calcium-activated potassium channels, and tandem pore domain potassium channels), and paxilline (PAX), which specifically blocks calcium-activated potassium channels. Paxilline is an indole diterpen that binds to KCa1.1 at an intracellular site that is involved in channel gating and seems to be coupled to the calcium binding site, whereas quinine blocks opening of KCa1.1 but also of other subtypes of potassium channels, including KCa3.1, Kv and K_2P_ [32,33,34,35,36].

Capacitation occurs within the female reproductive tract and consists of a series of changes that prepare sperm to fertilize the oocyte [21,22,23]. While plasma membrane integrity indicates that sperm are viable [37,38], its destabilization, which involves changes in its architecture and eventually leads to death, is one of the features of sperm capacitation [39]. The mechanisms underlying these changes are driven by a soluble adenylyl-cyclase, PKA-dependent signaling pathway (sAC-PKA), and are reliant upon the presence of cholesterol acceptors, such as proteins like bovine serum albumin (BSA) [40,41].

As expected, our results showed that whereas plasma membrane and acrosome integrity decreased throughout incubation with capacitated medium, the presence of 1 mM quinine, which inhibits several potassium channels, did partially counteract that reduction. This contrasted with the results of PAX, since at the highest concentration, this blocking agent of calcium-gated potassium channels reduced plasma membrane integrity. While these data suggest that quinine reduces the sperm ability to elicit in vitro capacitation and trigger progesterone-induced acrosome exocytosis, the increase in the percentages of spermatozoa with high membrane lipid disorder in samples incubated with 1 mM quinine does not seem to support that hypothesis. Again, the effects of PAX were the opposite, as the treatment with 1 mM PAX showed significantly lower percentages of spermatozoa with high membrane lipid disorder within the viable sperm population. Our results are in agreement with previous reports indicating that the inhibition of KCa1.1 channels, such as SLO1, prevents sperm capacitation in pigs without altering membrane lipid disorder [4]. Therefore, since quinine, which inhibits voltage-gated, calcium-activated, and tandem pore domain, potassium channels increased the lipid disorder of sperm plasma membrane, but the use of PAX, which specifically inhibits calcium-activated potassium channels did not have such an effect, we suggest that potassium channels other than the calcium-activated ones regulate this increase in the membrane lipid disorder, which is linked to cholesterol efflux, during in vitro capacitation. In addition to this, it is worth bearing in mind that quinine prevents the regulation of sperm volume in different species, including the pig [29]. Thus, one could suggest that alterations in the regulation of sperm volume could affect the architecture of sperm plasma membrane, which would be featured by an increase in membrane lipid disorder regulated by the aforementioned channels. Nevertheless, further research is needed to understand the precise relationship between potassium channels and membrane lipid architecture.

Capacitation is also characterized by changes in sperm motility, including hyperactivation [42]. When progesterone was added to control samples to induce acrosome exocytosis, we observed an increase in both total and progressive sperm motility. However, samples treated with quinine and 1 mM PAX did not exhibit that increase. In fact, the highest concentration of quinine (1 mM) significantly abolished sperm motility from the beginning of the experiment and until the end of the incubation period. This dramatic effect on sperm motility could be explained by the inability of sperm cells to regulate their volume [29,30]. In fact, our results are in agreement with previous studies in humans, which reported that quinine induces sperm to swell and that the resulting motility alterations lead sperm cells to fail to migrate and penetrate the cervical mucus [30]. In addition, even though not to the same extent as 1 mM quinine, the presence of 0.1 mM quinine and 1 mM PAX also decreased total and progressive motility. Therefore, our findings support the role of a wide variety of potassium channels, not only the calcium-activated but also the voltage-gated and the tandem pore domain potassium ones in the modulation of motility during sperm capacitation [33,43]. Remarkably, the fact that more than one type of channels could be involved in the modulation of sperm motility could not only explain why the effects observed were dose-dependent but also why the extent of the impact of quinine, which blocks many potassium channels, was larger than that of PAX.

An appropriate regulation of calcium influx is essential for mammalian sperm function, as motility, chemotaxis, capacitation, and acrosome reaction are governed by changes in intracellular levels of this secondary messenger [44,45]. Intracellular calcium has been reported to be stored in both the sperm head, specifically in the acrosomal region, and the region of the sperm neck and mid-piece in mammals, suggesting that head calcium could be involved in acrosomal exocytosis, and mid-piece calcium could be related to sperm motility and mitochondria energy production [46,47]. In the present study, these two calcium deposits were assessed by two separate fluorochromes: Fluo3, which has affinity for head and mid-piece stores, and Rhod5, which has more affinity for the head store. In both cases, we observed that intracellular calcium levels were significantly lower in treatments containing quinine and PAX, especially when added at the highest concentrations (i.e., 1 mM), than in the control. Hence, these results suggest that potassium channels, particularly the calcium-activated ones, play a pivotal role in regulating calcium influx during sperm capacitation in pigs. Moreover, progesterone is known to act as a potent acrosomal exocytosis inducer, rising intracellular calcium levels in both the sperm head and the mid-piece [48]. Herein, we found that while the addition of progesterone to control samples led to an increase in intracellular calcium levels, this rise was not as high as that found in the control when potassium channels were inhibited with 1 mM quinine or 1 mM PAX. Moreover, in control samples, intracellular calcium levels evaluated through Fluo3-staining were higher than those evaluated through Rhod5. Nevertheless, when potassium channels were inhibited with quinine or PAX, there was a much apparent decrease in the intracellular calcium levels stained by Fluo3, which targets the calcium residing in the mid-piece. This finding underpins the relevance of potassium-conductance in triggering calcium influx to the flagellum during in vitro sperm capacitation and progesterone-induced acrosome exocytosis. Moreover, CatSper channels in pigs have been shown to be essential for the regulation of sperm motility [25]. As aforementioned, not only does the general inhibition of potassium channels with quinine and the specific inhibition of calcium-activated potassium channels with PAX decrease calcium influx, but also reduces sperm motility. This suggest that, in pigs, calcium-activated potassium channels also regulate calcium influx by CatSper channels, reinforcing the hypothesis that potassium and calcium conductances are closely related.

Sperm mitochondria are located in the mid-piece and play an important role in maintaining appropriate energy levels for sperm function [49,50,51]. In control samples, mitochondrial membrane potential increased progressively during in vitro capacitation and after the induction of acrosome exocytosis with progesterone, which is in agreement with previous studies [47,52,53]. However, inhibiting potassium channels with quinine and PAX led sperm to exhibit lower MMP, especially at the highest quinine concentration. To the best of our knowledge, no previous study has investigated whether calcium-activated potassium channels are involved in the regulation of MMP during sperm capacitation in mammals. Since both the percentages and JC1_agg_/JC1_mon_ ratios of the sperm population with high MMP concurred in the same effect in the treatment containing 1 mM quinine but not in that with 1 mM PAX, our results suggest that in addition to calcium-activated potassium channels, the voltage-gated and the tandem pore domain potassium ones are involved in the regulation of mitochondrial activity during in vitro sperm capacitation. In this context, it is worth mentioning that, in bovine myocardium cells, quinine has also been reported to inhibit mitochondrial ATP-regulated potassium channel [54]. Therefore, the involvement of these mitochondrial channels could also contribute to explain the different effects observed in quinine- and PAX-blocked samples.

## 4. Materials and Methods

### 4.1. Reagents

Unless stated otherwise, all reagents were of analytic grade and were purchased from Sigma-Aldrich (Saint-Louis, MO, USA). Fluorochromes were acquired from Molecular Probes (Thermofisher Scientific; Waltham, MA, USA).

### 4.2. Semen Samples

A total of 15 semen samples, each coming from a separate boar, were used in this study. These samples were provided by a local farm (ServiciosGeneticosPorcinos, S.L.; Roda de Ter, Barcelona, Spain), which operates under standard, commercial conditions. Animals were housed in controlled conditions of temperature and humidity and fed with a standard and balanced diet; water was provided ad libitum. Ejaculates were collected twice a week through the gloved-hand method and the sperm-rich fraction was diluted to a final concentration of 30 × 10^6^ spermatozoa/mL with a commercial extender (Duragen, Magapor; Ejea de los Caballeros, Zaragoza, Spain). Diluted semen was cooled down to 17 °C and transported to the laboratory within four hours post-collection. All ejaculates fulfilled the following quality thresholds: >80% viable spermatozoa, 75% motile spermatozoa, and >80% morphologically normal spermatozoa.

Since authors did not manipulate any animal, but ejaculates were purchased from a commercial farm that operates under standard regulations, no specific authorization from an Ethics Committee was required. In addition, the aforementioned farm confirmed that they handle animals in accordance with the Animal Welfare Law issued by the Regional Government of Catalonia (Generalitat de Catalunya, Spain).

### 4.3. In Vitro Sperm Capacitation

Semen samples were centrifuged at 600× *g* at 17 °C for 10 min. Pellets were resuspended with capacitation medium to a final concentration of 20 × 10^6^ spermatozoa/mL. This capacitation medium consisted of 20 mM 4-(2-hydroxyethyl)-1-piperazineethanesulfonic acid (Hepes) buffer, 112 mM NaCl, 3.1 mM KCl, 5 mM glucose, 0.3 mM Na_2_HPO_4_, 0.4 mM MgSO_4_, 4.5 mM CaCl_2_, 21.7 mM L-lactate, 1 mM sodium pyruvate, 15 mM NaHCO_3_, and 5 mg/mL of bovine serum albumin (BSA). The osmolarity was 305 ± 7 mOsm/Kg, and the pH was adjusted to 7.4. Sperm were incubated at 38.5 °C and 5% CO_2_ for 300 min in a Heracell 150 incubator (Heraeus Instruments GmbH, Osterode, Germany), as described in Rocco et al. [55]. After 240 min, progesterone was added to a final concentration of 10 µg/mL to induce the acrosome exocytosis. At the beginning of the experiment, after 120 min and 240 min of incubation, and after 5, 30 min, and 60 min of progesterone addition (i.e., 245, 270 min, and 300 min), an aliquot was taken to evaluate sperm motility, plasma membrane and acrosome integrity, membrane lipid disorder, intracellular calcium levels, and mitochondrial membrane potential.

### 4.4. Inhibition of Calcium-Activated Potassium Channels with Quinine and Paxilline

In addition to the control, which resulted from incubating sperm with capacitating medium, sperm samples, previously resuspended in the same medium, were added with 0.1 quinine, 1 mM quinine, 0.1 mM PAX, or 1 mM PAX. These concentrations were set based on previous studies [4,11,30,56] and preliminary experiments conducted in our laboratory in a 10-fold series. The two chosen concentrations were the lowest one at which significant differences with regard to the control were observed, and the highest one that showed the clearest effect without being cytotoxic. Sperm were incubated for 300 min and added with 10 µg/mL progesterone after 240 min of incubation, as described previously.

### 4.5. Flow Cytometry Analyses

Information on flow cytometry experiments is provided following the recommendations of the International Society for Advancement of Cytometry (ISAC) [57]. Flow cytometry was used to evaluate the integrities of plasma membrane and acrosome, membrane lipid disorder, intracellular calcium levels, and mitochondrial membrane potential. Prior to staining, sperm concentration was adjusted to 1 × 10^6^ spermatozoa/mL, as described in Yeste et al. [58]. Sperm cells were subsequently stained with the appropriate combinations of fluorochromes, as described below.

Spermatozoa were excited through an argon ion laser (488 nm; power: 22 mW) using a Cell Laboratory Quanta^TM^ SC cytometer (Beckman Coulter, Fullerton, CA, USA). In this equipment, electronic volume (EV) of particle is evaluated using the Coulter principle and the forward scatter (FS) is replaced by the EV. In addition, the EV channel was periodically calibrated using 10-µm Flow-Check fluorospheres (Beckman Coulter) by positioning this size of the bead at channel 200 on the volume scale. Three different optical filters were used to evaluate sperm samples; these filters had the following characteristics: FL1 (green fluorescence): Dichroic/splitter long pass, DRLP: 550 nm, band pass, BP: 525 nm, detection width: 505–545 nm; FL2 (orange fluorescence): DRLP: 600 nm, BP: 575 nm, detection width: 560–590 nm); FL3 (red fluorescence): Long pass, LP: 670 nm, detection width: 655–685 nm. Signals were logarithmically amplified, and photomultiplier settings were adjusted to particular staining methods. FL1 was used to detect green fluorescence (YO-PRO-1; Fluo3; and JC1 monomers, JC1_mon_), FL2 was utilized to detect JC1 aggregates (JC1_agg_) and FL3 was used to detect red fluorescence (Merocyanine-540, M540; and Rhod5). Sheath flow rate was set at 4.17 µL/min in all analyses, and EV and side-scatter (SS) were recorded in a linear mode (in EV vs. SS dot plots) for a minimum of 5000 events per replicate. The analyzer threshold was adjusted on the EV channel to exclude subcellular debris (particle diameter < 7 µm) and cell aggregates (particle diameter >12 µm). Therefore, sperm-specific events were positively gated on the basis of their EV/SS distribution, whereas the others were gated out. In some protocols, compensation was used to minimize spill-over of green fluorescence into the red channel, as described below. Dot-plots (FL1 vs. FL3; FL2 vs. FL3) were analyzed through Cell Lab QuantaSC MPL Analysis Software (version 1.0; Beckman Coulter). In addition, data obtained from flow-cytometry experiments were corrected according to the procedure described by Petrunkina et al. [59], using the percentage of debris particles detected in the SYBR14/PI staining (see Section 4.5.1). Each sample and parameter were evaluated in triplicate using independent tubes, and the mean ± SEM was subsequently calculated.

#### 4.5.1. Plasma Membrane Integrity

Plasma membrane integrity was evaluated using the LIVE/DEAD^®^ sperm viability kit (Molecular Probes, Thermo Fisher Scientific; Waltham, MA, USA). Briefly, spermatozoa, previously diluted to a final concentration of 1 × 10^6^ sperm/mL, were stained with SYBR14 (final concentration: 100 µM) for 10 min at 38 °C in the dark. Following this, spermatozoa were incubated with propidium iodide (PI; final concentration: 12 µM) for 5 min at the aforementioned conditions. Fluorescence of SYBR14 was detected through FL1, whereas that of PI was collected through FL3. Each spermatozoon was classified as with an intact (SYBR14^+^/PI^−^, green) or non-intact plasma membrane (SYBR14^+^/PI^+^, orange; or SYBR14^−^/PI^+^, red). Unstained and single-stained samples were used for setting the EV-gain, and FL1 and FL3 PMT voltages. Spillover from FL1 into the FL3 channel was compensated (2.45%). Percentages of non-sperm, debris particles appearing in the lower left quadrant (SYBR14^−^/PI^−^) were used to correct percentages of particles corresponding to sperm in this and other tests.

#### 4.5.2. Acrosome Integrity

Acrosome integrity was evaluated following the protocol set by Cooper and Yeung [60] and adapted to pig spermatozoa by Rocco et al. [55]. In this protocol, sperm are first stained with ethidium homodimer (3,8-diamino-5-ethyl-6-phenylphenanthridinium bromide; EthD-1), then permeabilized and finally stained with the lectin from *Arachis hypogaea* (peanut agglutinin, PNA) conjugated with fluorescein isothiocyanate (FITC). Briefly, samples were incubated with EthD-1 (final concentration: 2.5 µg/mL) at 38 °C for 5 min in the dark. Following centrifugation at 2000× *g* and 17 °C for 30 s, spermatozoa were resuspended with PBS containing BSA (4 mg/mL). Thereafter, spermatozoa were again centrifuged at 2000× *g* and 17 °C for 30 s; the pellet was resuspended with 100 µL of ice-cold 100% methanol and incubated for 30 s. After centrifugation at 2000× *g* and 17 °C for 30 s, samples were resuspended with 250 µL PBS, and then added with 0.8 µL PNA-FITC (final concentration: 2.5 µM). Samples were incubated at 25 °C for 15 min in the dark and then centrifuged at 2000× *g* and 17 °C for 30 s. Pellets were resuspended with 0.6 µL PBS and centrifuged 2000× *g* and 17 °C for 30 s. This step was repeated two times. Finally, spermatozoa were evaluated with the flow cytometer and classified into one of the following populations: (1) Viable spermatozoa with an intact acrosome (PNA^+^/EthD-1^−^); (2) viable spermatozoa with an exocytosed acrosome (PNA^−^/EthD-1^−^); (3) non-viable spermatozoa with an intact acrosome (PNA^+^/EthD-1^+^); and (4) non-viable spermatozoa with an exocytosed acrosome (PNA^−^/EthD-1^+^). FL3 was used to detect red fluorescence from EthD-1, and FL1 to detect that of PNA-FITC. Unstained and single-stained samples were used for setting the EV gain, FL1 and FL3 PMT-voltages, and for compensation of FL1 spill over into the FL3 channel (2.70%). Percentages of PNA^−^/PI^−^spermatozoa were corrected with the percentages non-sperm debris particles (SYBR14^−^/PI^−^) and the other sperm proportions were recalculated.

#### 4.5.3. Membrane Lipid Disorder

Membrane lipid changes were determined through staining with Merocyanine-540 (M540) and YO-PRO-1, as described by Harrison et al. [61]. In brief, spermatozoa were incubated with M540 (final concentration: 2.6 µM) and YO-PRO-1 (final concentration: 25 nM) at 38 °C for 10 min in the dark. A total of four sperm populations were identified: (1) Non-viable spermatozoa with low membrane lipid disorder (M540^−^/YO-PRO-1^+^), (2) non-viable spermatozoa with high membrane lipid disorder (M540^+^/YO-PRO-1^+^), (3) viable spermatozoa with low membrane lipid disorder (M540^−^/YO-PRO-1^−^), and (4) viable spermatozoa with high membrane lipid disorder (M540^+^/YO-PRO-1^−^). Fluorescence from M540 was detected through FL3, and that from YO-PRO-1 was collected through FL1. Unstained and single-stained samples were used for setting the electronic volume (EV) gain, FL1, and FL3 PMT-voltages. Data were not compensated. Percentages of debris particles found in SYBR14/PI staining (SYBR14^−^/PI^−^) were subtracted from the percentages of M540^−^/YO-PRO-1^−^spermatozoa; the percentages of the other sperm populations were recalculated.

#### 4.5.4. Intracellular Calcium Levels

Intracellular calcium levels of spermatozoa were determined by using two different fluorochromes, Fluo3 and Rhod5. On the one hand, Fluo3 staining was performed following the protocol described by Harrison et al. [62] and modified by Kadirvel et al. [63]. Briefly, spermatozoa were incubated with Fluo3-AM (final concentration: 1 µM) and PI (final concentration: 12 µM) at 38 °C for 10 min in the dark. Fluorescence emitted by Fluo3 was collected through FL1, whereas that of PI was detected with FL3. When samples were analyzed with the flow cytometer, four populations were identified: (1) Viable spermatozoa with low intracellular calcium levels (Fluo3^−^/PI^−^); (2) viable spermatozoa with high intracellular calcium levels (Fluo3^+^/PI^−^); (3) non-viable spermatozoa with low intracellular calcium levels (Fluo3^−^/PI^+^); and (4) non-viable spermatozoa with high intracellular calcium levels (Fluo3^+^/PI^+^). Unstained and single-stained samples were used for setting the EV-gain, FL1 and FL3 PMT voltages, and for compensating FL1 spill over into the FL3-channel (2.45%) and FL3 spill over into the FL1-channel (28.72%). Percentages of debris particles found in SYBR14/PI staining (SYBR14^−^/PI^−^) were subtracted from the percentages of Fluo3^−^/PI^−^spermatozoa; the percentages of the other sperm populations were recalculated.

On the other hand, intracellular calcium levels were also evaluated with Rhod5 labelling, following the protocol described by Yeste et al. [47]. Spermatozoa were incubated with Rhod5N-AM (final concentration: 5 µM) and YO-PRO-1 (final concentration: 25 nM) and incubated at 38 °C for 10 min in the dark. Rhod5 fluorescence was collected through FL3, whereas that of YO-PRO-1 was detected by FL1. Spermatozoa were classified into one of the following categories: (1) Viable spermatozoa with low intracellular calcium levels (Rhod5^−^/YO-PRO-1^−^); (2) viable spermatozoa with high intracellular calcium levels (Rhod5^+^/YO-PRO-1^−^); (3) non-viable spermatozoa with low intracellular calcium levels (Rhod5^−^/YO-PRO-1^+^); and (4) non-viable spermatozoa with high intracellular calcium levels (Rhod5^+^/YO-PRO-1^+^). Unstained and single-stained samples were used for setting the EV-gain, FL1 and FL3 PMT voltages, and for compensating FL3 spill over into the FL1-channel (3.16%) and FL3 spill over into the FL1-channel (28.72%). Percentages of debris particles found in SYBR14/PI staining (SYBR14^−^/PI^−^) were subtracted from the percentages of Rhod5^−^/YO-PRO-1^−^spermatozoa; the percentages of the other sperm populations were recalculated.

#### 4.5.5. Mitochondrial Membrane Potential

Mitochondrial membrane potential (MMP) was determined following the protocol described by Guthrie and Welch [64]. Spermatozoa were incubated with JC1 (5,5′,6,6′-tetrachloro-1,1′,3,3′tetraethylbenzimidazolylcarbocyanine iodide) at room temperature for 30 min the dark. JC1 molecules remain as monomers (JC1_mon_) at low MMP, and form aggregates (JC1_agg_) when they detect high MMP. Two different emission filters (FL1 and FL2) were used to differentiate two sperm populations: (a) Spermatozoa with high MMP (JC1_agg_), and (b) spermatozoa with low MMP (JC1_mon_). Percentages of spermatozoa with high MMP corresponded to the orange-stained spermatozoa, which appeared in the upper half of the diagram in FL1 vs. FL2 dot-plots. FL1 spill-over into the FL2 channel was compensated (51.70%). Percentages of debris particles found in SYBR14/PI staining (SYBR14^−^/PI^−^) were subtracted from the percentages of spermatozoa appearing in the lower left part of the diagram; percentages of the two sperm populations were recalculated.

### 4.6. Analysis of Sperm Motility

Sperm motility was assessed through a computerized assisted sperm analysis (CASA) system (ISAS version 1.2; Proiser R+D, Valencia, Spain) under a phase-contrast microscope (Olympus BX41; Olympus Europa GmbH, Hamburg, Germany) at 100× magnification. Following this, 5 µL of each sample was placed onto a pre-warmed (38 °C) Makler counting chamber (Sefi-Medical Instruments; Haifa, Israel) and a minimum of 1000 spermatozoa per replicate were evaluated. The analyzed parameter ranges were: Curvilinear velocity (VCL), which is the mean path velocity of the sperm head along its actual trajectory (µm/s); linear velocity (VSL), which is the mean path velocity of the sperm head along a straight line from its first to its last position (µm/s); mean velocity (VAP), which is the mean velocity of the sperm head along its average trajectory (µm/s); linearity coefficient (LIN), which results from VSL/VCL × 100 (%); straightness coefficient (STR), which results from VSL/VAP × 100 (%); wobble coefficient (WOB), which results from VAP/VCL × 100 (%); mean amplitude of lateral head displacement (ALH), which is the mean value of the extreme side-to-side movement of the sperm head in each beat cycle (µm); and frequency of head displacement (BCF), which is the frequency with which the actual sperm trajectory crosses the average path trajectory (Hz). A spermatozoon was considered to be motile when its VAP was ≥10 µm/s, and progressively motile when STR was ≥45%.

### 4.7. Statistical Analyses

Statistical analyses were performed using IBM SPSS 25.0 for Windows (IBM Corp., Armonk, NY, USA). Sperm quality and function parameters (plasma membrane and acrosome integrity, membrane lipid disorder, intracellular calcium levels, mitochondrial membrane potential, and sperm motility) were considered as dependent variables, whereas each experiment and incubation treatments using seminal samples from different boars were treated as biological replicates. All the variables were first tested for normality (Shapiro–Wilk test) and homoscedasticity (Levene test); when needed, data (x) were transformed with arcsin √x. Results were then evaluated with a mixed model (intra-subject factor: Incubation time; inter-subject factor: Treatment) followed by the post-hoc Sidak test for pair-wise comparisons. Because each sperm sample came from a separate boar, the animal could not be included as a random-effects factor in the model. The level of significance was set at *p* ≤ 0.05 in all cases.

## 5. Conclusions

In conclusion, our results indicate that potassium channels are essential for sperm to elicit sperm capacitation and trigger acrosome exocytosis. Specifically, blocking potassium channels with quinine prevents sperm to achieve the capacitated status by decreasing their motility and mitochondrial function. Moreover, inhibition of these channels reduces calcium influx to both the sperm head and the mid-piece and hinders sperm to trigger acrosome exocytosis. However, in the case of quinine, these effects were unexpectedly observed together with a concomitant increase in membrane lipid disorder. On the other hand, quinine-induced effects, which targets a wide range of potassium channels, were larger than those observed with PAX, a specific blocker of calcium-activated potassium channels. While this indicates that potassium channels, other than the calcium-activated ones, which comprise voltage-gated, tandem pore domain, and mitochondrial ATP-regulated potassium channels are involved in sperm capacitation, further research should address how these channels alter the cAMP/PKA signaling pathway related to sperm capacitation, and the relationship of calcium-activated potassium channels with other ion channels, including CatSper.

## Data Availability

The data presented in this study are available on request from the corresponding author.

## Supplementary Materials

The following are available online at https://www.mdpi.com/1422-0067/22/4/1992/s1, Figure S1: (a) Percentages of viable spermatozoa with an intact acrosome (PNA^+^/EthD-1^−^) and (b) percentages of viable spermatozoa with low membrane lipid disorder (M540^−^/YO-PRO-1^−^) during in vitro capacitation and progesterone-induced acrosomal exocytosis (300 min) in the presence of 0.1 mM quinine, 1 mM quinine, 0.1 mM paxilline (PAX) and 1 mM PAX. Black arrow indicates the time at which 10 μg/mL progesterone was added to induce acrosomal exocytosis (i.e., 240 min). Different letters (a-c) mean significant (*p* < 0.05) differences between treatments at a given time point. Data are shown as mean ± SEM for 15 independent experiments; Figure S2: Representative dot-plots for M540/YO-PRO-1 staining in control and quinine- and PAX-blocked samples; Figure S3: Representative dot-plots for JC1 staining in control and quinine- and PAX-blocked samples.

## Abbreviations

ALH	Amplitude of Lateral Head Displacement
BCF	Beat Cross Frequency
BSA	Bovine Serum Albumin
CASA	Computer-Assisted Sperm Analysis
CFTR	Cystic Fibrosis Transmembran Conductance Regulator
CM	Capacitating Medium
DANCE	Forward Movement of the Head
EV	Electronic Volume
FITC	Fluorescein Isothiocyanate
FSC	Forward Scatter
GSK3a	Glycogen Synthase Kinase-3 isoform α
GSK3ß	Glycogen Synthase Kinase-3 isoform β
ISAC	International Society for Advancement of Cytometry
ISAS	Integrated Sperm Analysis System
JC1	5,5′,6,6′-tetrachloro-1,1′,3,3′tetraethylbenzimidazolylcarbocyanine iodide
LIN	Linearity
M540	Merocyanine-540
MAD	Mean Angular Displacement
MMP	Mithocondrial membrane potential
PCA	Principal Component Analysis
PI	Propidium Iodide
PMOT	Progressive Motility
PNA	Peanut Agglutinin
SEM	Standard Error of the Mean
SP	Motile subpopulation
SRF	Sperm-Rich Fraction
SSC	Side Scatter
STR	Straightness
TMOT	Total Motility
VAP	Average Path Velocity
VCL	Curvilinear Velocity
VSL	Straight Linear Velocity
WOB	Wobble Coefficient
