# Supplementary figures and images for: Inhibition of Potassium Channels Affects the Ability of Pig Spermatozoa to Elicit Capacitation and Trigger the Acrosome Exocytosis Induced by Progesterone
Source: Int J Mol Sci. 2021 Feb 17;22(4):1992. doi: 10.3390/ijms22041992 (PMC7922121; doi:10.3390/ijms22041992)

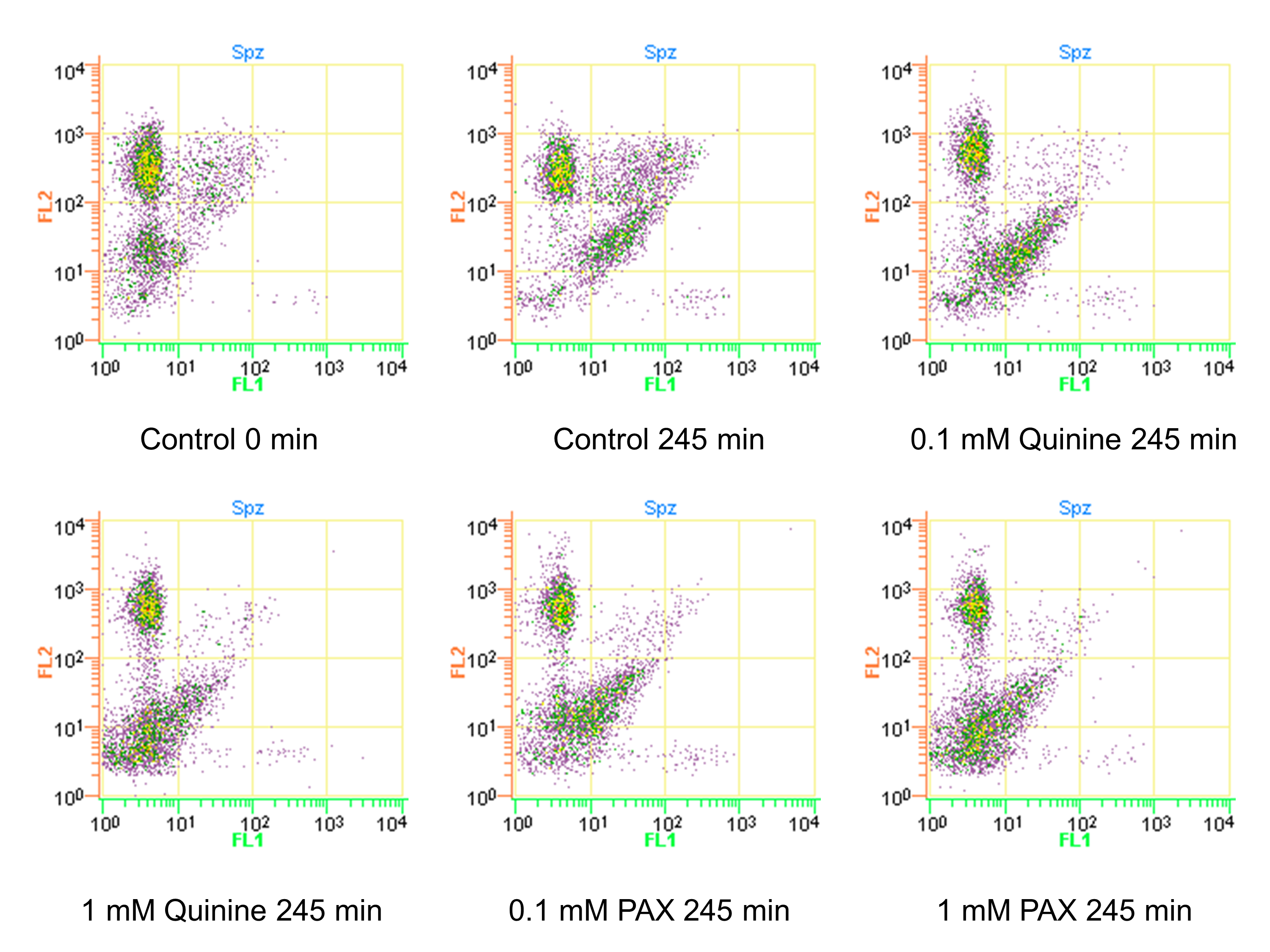

Supplement: Supplementary file 1 [file ijms-22-01992-s001.zip › Suppl Fig S3.TIF]

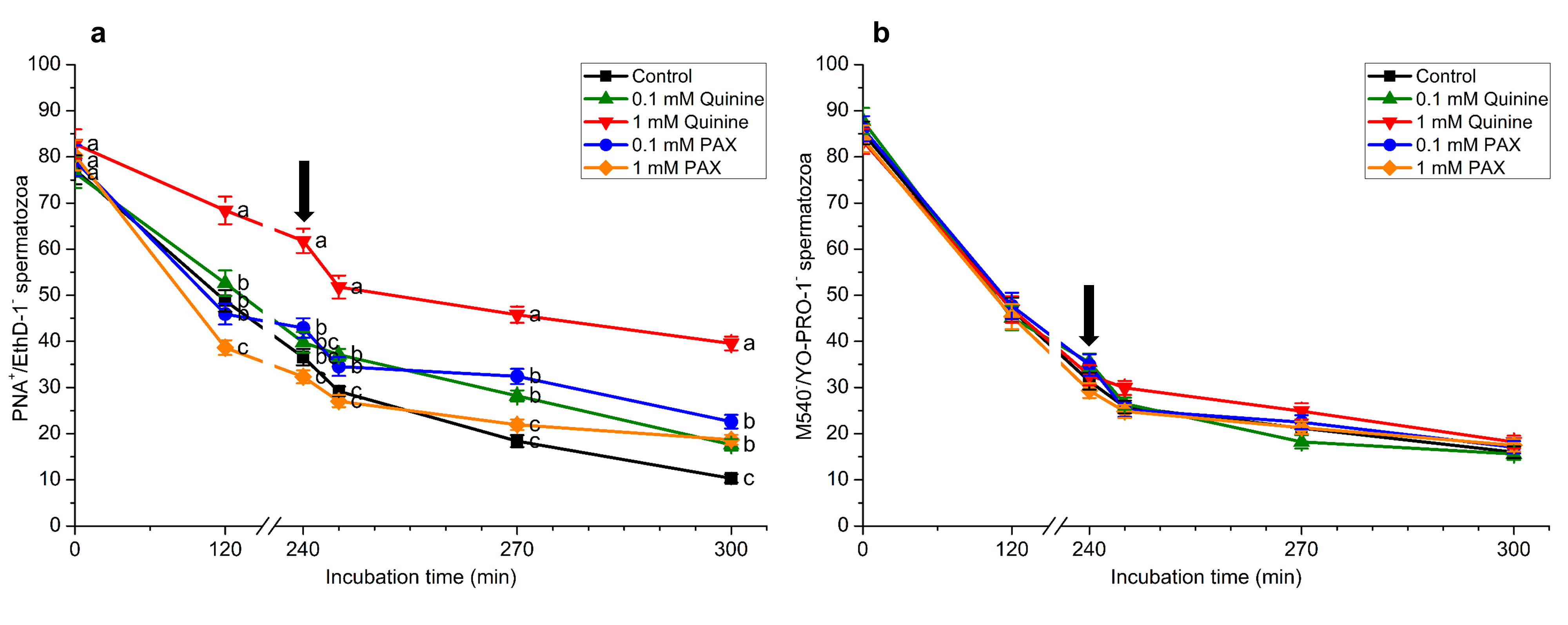

Supplement: Supplementary file 1 [file ijms-22-01992-s001.zip › Suppl Fig S1_PNA_M540_Noto_et_al_Quinine rev2.tif]

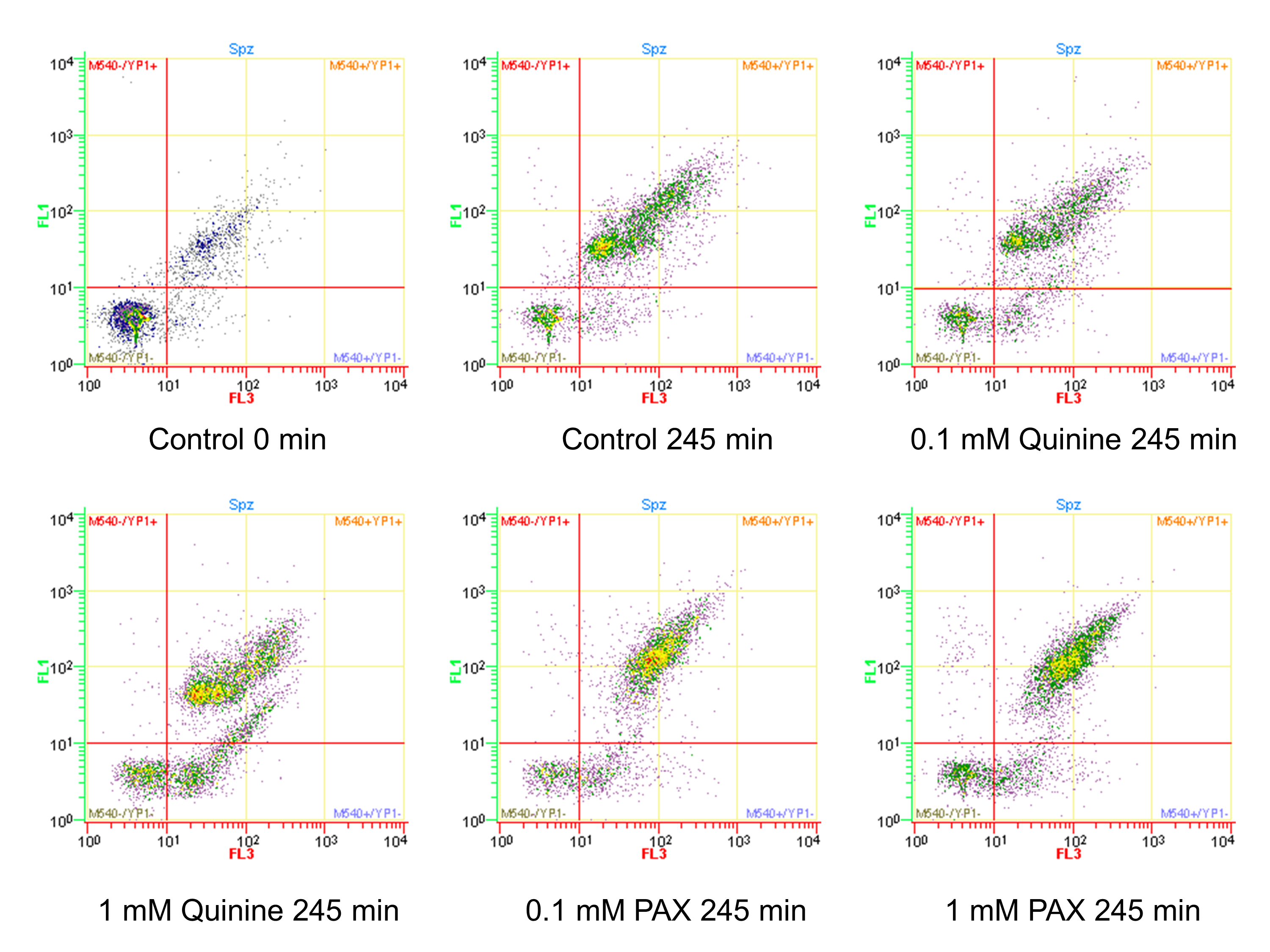

Supplement: Supplementary file 1 [file ijms-22-01992-s001.zip › Suppl Fig S2.TIF]
